# Irradiation deformation near different atomic grain boundaries in α-Zr: An investigation of thermodynamics and kinetics of point defects

**DOI:** 10.1038/srep23333

**Published:** 2016-03-23

**Authors:** A. Arjhangmehr, S. A. H. Feghhi

**Affiliations:** 1 Department of Radiation Application, Shahid Beheshti University G.C, Tehran, IRAN

## Abstract

Understanding radiation performance of nanocrystalline Zr-based alloys is essential to develop internal components and external cladding materials with self-healing capabilities for longer and safer life cycles in harsh reactor environments. However, the precise role of interfaces in modifying defect production and evolution in α-Zr is not yet determined. Using atomistic simulation methods, we investigate the influence of different atomic grain boundaries (GBs) in thermodynamic and kinetic properties of defects on short timescales. We observe that the sink efficiency and sink strength of interfaces vary significantly with the boundary structures, with a preference to absorb interstitials (vacancies) when the GBs are semi-parallel (semi-perpendicular) relative to the basal planes. Further, we identify three distinct primary cascade geometries, and find that the residual defect clustering in grain interiors depends on how the atomic GBs modify the spatial distribution of defects within the crystal structure. Finally, we explain and discuss the dynamic results in terms of energetic and kinetic behaviors of defects near the pristine and damaged boundaries. Eventually, these will provide a microscopic reference for further improving the radiation response of Zr by using fine grains or by introducing a high density of dispersoids in material metallurgy.

The development of zirconium (Zr) metallurgy is essentially due to the nuclear industry, where zirconium alloys have come to be regarded as the proven structural materials^1^. The unique properties of zirconium, such as excellent corrosion resistance at 300 °C^2^, higher thermal conductivity and superior dimensional stability at elevated temperatures in comparison with stainless steel alloys^1^, good mechanical properties and low capture cross section (good neutron economy)^2^ have made zirconium and its alloys (Zircaloy-2, Zircaloy-4 and Zr-2.5%Nb) the ideal cladding materials to encase the uranium dioxide (UO_2_) fuel pellets in Pressurized Water Reactors (PWR)^1^ and Boiling Water Reactors (BWR)^1^, and to be used as the main structural component of calandria^1^ and pressure^2^ tubes in CANadian Deuterium Uranium (CANDU) reactors^2^. Moreover, the various zirconium alloy grades used in light and heavy water nuclear reactors are also available for nuclear waste disposal containers as internal components or external cladding to ensure the safe containment throughout the time required for the radioactivity decay to safe levels^2^. Additional advantages of zirconium alloys for long term nuclear waste disposal include excellent radiation stability and 100% compatibility with existing Zircaloy fuel cladding to alleviate any concerns of galvanic corrosion^2^.

In fission reactors and nuclear waste disposal, zirconium and Zr-based alloys experience severe irradiation environments^3^. The exposure to extremely high heat^3^, high neutron and gamma doses^4^,^5^, and high energy product particles resulting from neutron-solid interactions^6^ will induce microstructure and microchemistry changes, due to the atomic displacements and formation of point defects and defect clusters (e.g., the formation of <*a*> dislocation loops, <*c*> component dislocation loops and cavities (voids and bubbles)^7^,^8^,^9^,^10^,^11^,^12^,^13^,^14^,^15^,^16^,^17^,^18^,^19^,^20^,^21^,^22^). These will lead to various structural failures including growth, hardening, creep, corrosion and amorphization^20^,^21^,^22^,^23^,^24^, which in turn, will impose severe limitations on possible operating parameters in terms of neutron and gamma doses and allowable stresses and temperatures. For nuclear usage, it is necessary for zirconium and zirconium-based alloys to not only withstand radiation damage, but also keep their intrinsic thermal properties and mechanical stability to assure long-term material performance during reactor operation and waste containment.

Toward development of radiation tolerant materials, previous studies have shown that the nano-structured cubic metals with tailored microstructures, i.e. interface-dominant structures with nano-sized grains can enhance the radiation tolerance of the irradiated materials, since the grain boundaries (GBs) can serve as sinks for absorbing and annihilating radiation-induced defects^25^,^26^,^27^,^28^,^29^,^30^,^31^,^32^,^33^,^34^,^35^,^36^. *Samaras et al.*^37^,^38^ showed that the GBs in nano-crystalline face-centered cubic (*fcc*) and body-centered cubic (*bcc*) structures act as sinks for self-interstitial atoms (SIA), and deduced that for *fcc* Ni the absorption of interstitials leads to increasing production of stacking-fault tetrahedral, which they related to the dislocation structure of the GBs. *Feghhi et al.* investigated the influence of atomic GBs on the energetic and kinetic properties of defects in *bcc* Fe^39^ and *fcc* Ni^40^, and reported substantially large reduction of the defect formation energy and vacancy migration barrier in the neighboring region of the GBs, which they considered as the driving forces to observe biased absorption of interstitials over vacancies^39^,^40^. *Tooq et al.*^41^ studied defects evolution in *fcc* Ni with different GB structures and determined that the trapped vacancies in grain interiors had a tendency to cluster, and were immobile on picosecond (ps) timescale. *Zeng et al.*^42^ reported that the vacancy cluster size distribution shifted to larger sizes, when there was only a limited overlap between the cascade and the GB, in which most of the radiation-produced defects formed in the bulk grain^42^. *Bai et al.*^43^,^44^ investigated the radiation tolerance of *fcc* Cu with different atomic GBs and concluded that while there were features generic to most of these structures, significant differences also existed that must be accounted for to accurately predict the evolution of the material under irradiation.

In the case of hexagonal close-packed (*hcp*) materials, *Yao*^45^ investigated dynamics of defects in *hcp* Ti, and reported that the GBs act as sinks of radiation-produced point defects, with efficiency depending on the degree of overlap between the GB and the cascade damage region. *Solanki and Bhatia*^46^ investigated the defect energetics near 190 symmetric tilt GBs with different 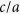 ratios in *hcp* Ti, Mg and Zr. They observed that the local arrangements of GBs and the resulting structural units had a significant influence on the magnitude of vacancy binding energies^46^. Also, they reported that there were atoms lying symmetrically along the GB plane that had vacancy binding energies close to or even higher than the bulk values, and predicted that the GBs may not provide pathways for vacancy diffusion^46^. *Woo* in the theory of irradiation deformation in non-cubic metals^47^, reported that SIAs had a higher mobility in the basal plane than along the <c> axis in *hcp* Zr and that the vacancies had semi-isotropic diffusional behavior. So, he concluded that the GBs perpendicular to the basal plane absorbed more interstitial atoms, while GBs parallel to the basal plane absorbed more vacancies^47^. Thus, contrary to the implications of the conventional rate theory and previous reports in cubic materials, he predicted that the GBs in α-Zr were not necessarily biased toward SIAs, and were no longer neutral sinks^47^.

To date, the atomistic studies of defect-boundary interaction mechanisms in relation to radiation damage in *hcp* materials are still limited and the precise role of interfaces in modifying defect production and evolution in α-Zr is not yet determined. Therefore, a systematic study of the influence of different atomic GBs on defect dynamics, energetics and kinetics in α-Zr is warranted to shed light on these processes at the atomistic levels. In this study, we examine four GBs in *hcp* Zr with different atomic structures and determine the role that they play in modifying defect production and evolution on ps timescale. We observe that the twist boundaries are defect sinks with a preference for interstitials, when the cascades centers slightly overlap with the GBs and most of the defects are produced in the bulk grains. Moreover, we find that the sink efficiency and the sink strength of the symmetric tilt structures depend on the crystallographic orientation of the grains within the bi-crystal structures, acting as strong defect sinks for SIAs when the GBs are semi-parallel to the basal planes and weak absorbers with a preference for vacancies when the GBs are semi-perpendicular.

Then, we discuss the mechanisms that lead to the aggregation of defects (interstitial or vacancy) near the boundaries, through the analysis of the spatial distribution of defects within the primary cascades. We identify three distinct defect distributions based on our molecular dynamic simulations, denoted as semi-spheroid, semi-ellipsoid and fragmented cascades, and find that the clustering of residual vacancies and interstitials in the grain interiors depends on how the atomic GBs modify the spatial distribution of defects within the bi-crystal structures. Further, we investigate how the different GB structures influence the primary damage cascades, emphasizing on the energetics and kinetics of defects in the neighboring region of the pristine and damaged boundaries. We find that the defect formation energies and migration barriers are significantly reduced near the boundaries, indicating that the GBs facilitate the formation and subsequent diffusion of defects toward the GB center. Meanwhile, we explain the dynamic results in terms of the energetic and kinetic behaviors of the radiation-produced vacancies and interstitial atoms. Eventually, these will provide a microscopic reference for further improving the radiation performance of Zr by using fine grains or by introducing a high density of dispersoids.

## Method

**Figure 1:**
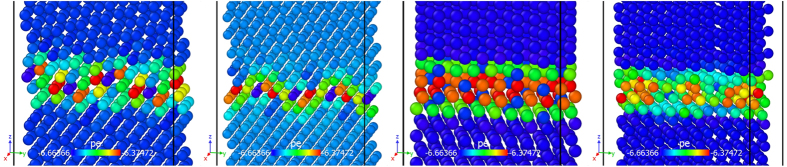
Structure of four atomic GBs considered here: (**a**) symmetric tilt 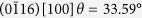; (**b**) symmetric tilt 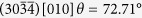; (**c**) twist 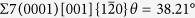; (**d**) twist 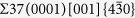 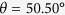. In each figure, the structure is shown perpendicular to the grain boundary normal. The atoms are colored based on their potential energies (pe). In each GB structure, the axes of the reduced coordinates are: 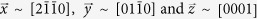.

In this paper, we investigate the influence of different atomic GBs on energetic, thermodynamic and kinetic properties of radiation-produced defects in *hcp* Zr, using molecular statics (MS), molecular dynamics (MD) and the climbing image-nudged elastic band (climbing-NEB) method. In particular, we are interested in how atomic GBs modify the evolution and spatial distribution of point defects and defect clusters in grain interiors. To determine how the presence of GBs influences the primary damage stage, we have investigated the interaction of point defects with the pristine boundaries^40^. To do so, we have created four bi-crystal structures, including symmetric tilt 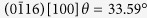 (low-angle symmetric tilt; semi-parallel relative to the basal plane), symmetric tilt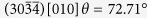 (high-angle symmetric tilt; semi-perpendicular relative to the basal plane) and twist 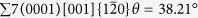, twist 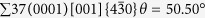 (parallel relative to the basal plane), which represent the complex nature of GBs in *hcp* materials. The atomic coordinates for the grain boundary structures are generated using GB studio^48^. In order to release stress in the GB structures, we have used steepest descent minimization method^39^,^40^ under zero external pressure to obtain the minimum energy configurations of four GB structures considered here, as shown in Fig. 1. The MS and MD simulations are performed by using Large-scale Atomic Molecular Massively Parallel Simulator (LAMMPS)^49^.

In order to accurately describe Zr-Zr interactions, embedded-atom method (EAM) potential developed by *Mendelev and Ackland* (*#3*)^50^, which properly describes the *hcp* to *bcc* phase-transition and liquid-structure data, is employed. This potential provides a better description of stacking fault energy than previous ones^51^. The potential gives a vacancy formation energy of 1.72 ± 0.05 eV and diffusion barrier of 0.62 ± 0.04 eV (0. 71 ± 0.06 eV) for migration of a vacancy in basal plane (non-basal plane) and the interstitial configuration of “octahedral site” with a formation energy of 2.82 ± 0.02 eV and migration barrier of 0.013 ± 0.01 eV at 0 K, which is in close agreement with the recent DFT calculations regarding anisotropy behavior of defects in *hcp* Zr^52^. The calculated and analyzed properties include: “*the relative number of residual defects in bulk region*”, “*vacancy/interstitial cluster analysis in neighboring region of the GBs*”, “*analysis of the spatial distribution of defects within the primary cascades*”, and “*vacancy and interstitial formation energies and diffusion barriers near pristine and damaged GBs*”. Through the calculation of these energetic and kinetic properties, we can have a better understanding of the influence of different atomic GB structures on the defect-boundary interaction^40^.

For MD simulations, we have used the same method proposed in our previous report in^40^ that includes a block with slab geometry. To simulate and study the behavior of primary damage cascades, two systems are constructed^34^,^35^,^36^,^37^,^38^,^39^,^40^,^42^,^44^. The large system consisting of 642784–687452 atoms with a size of about ~241.3 × 242.2 × 264.9 Å^3^ is used for simulating the primary radiation damage in Zr with GBs. The small system having 3890–4140 atoms with a size of about ~21.1 × 22.6 × 80.9 Å^3^ is used for calculating the energetic and kinetic properties of point defects. Periodic boundary conditions are applied in the directions parallel to the GB plane, and a fixed boundary condition in the direction normal to the GB plane^40^. In this configuration, the free atoms are sandwiched between two slabs of fixed atoms on each side of the GB plane^39^,^40^,^53^. Since the fixed boundary condition is applied in the direction normal to the GB, the size of the system in this direction is chosen to avoid interactions between the fixed boundary and the primary cascade^40^,^53^. Also, we have carefully checked that the sizes of the systems are large enough to avoid interactions between two neighboring cells, and a bulk region always exists between the fixed boundary and the GB^40^,^43^,^53^. The atoms in the outmost three layers of the moving region are coupled with a velocity-rescaling thermostat to maintain the system temperature at 300 K during the initiation and subsequent relaxation of the primary cascade.

An atom, at different distances from the GB plane, is chosen as the PKA with 3, 6 and 9 keV of kinetic energy, directed perpendicularly towards the GB^39^,^40^,^53^. The final damaged structure is compared to the undamaged GB structure relaxed at 300 K (the reference system) to characterize defects, using the Wigner-Seitz (WS) cell method^39^,^40^,^53^. In the WS method, the whole system is divided into polyhedral WS cells centered on lattice sites. If a polyhedral WS cell contains no atom, the lattice site is labeled a vacancy and if the cell infolds more than one atom, it is considered as a self-interstitial atom (SIA)^40^. The width of the GB is defined as the region near the dividing interface, which includes atoms with potential energies outside the interval 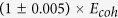
40, where E_coh_ ~6.47 eV is the cohesive energy of a Zr atom according to the potential used. Only the defects remaining in the bulk region are counted as surviving defects, and two vacancies (interstitials) are considered to belong to the same cluster, if their separation is less than 3.16 ~ *a* (*hcp*) Å. In each cluster, only the net number of defects is counted. To have good statistical approximation, at each PKA distance, 8 cascade simulations are performed. Results are compared to cascades in a single crystal with the same PKA energy and direction relative to the crystallographic orientation as in the cascade simulations for each GB^39^,^40^. The visualization of the primary cascades is performed using OVITO^54^.

To calculate the defect formation energies, we follow the method proposed in^43^,^53^. Initially, the created cell is structurally relaxed at 0 K, using steepest descent minimization method. Then, an interstitial or a vacancy is created in the region of interest and the second minimization is performed, to allow the point defect find its stable location^40^. The formation energies of point defects are calculated by


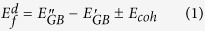


where 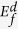 is the formation energy of a defect (the positive sign corresponds to the vacancy and the negative sign corresponds to the interstitial), 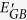 (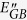) is the total potential energy of the system without (with) a defect, and 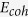 is the cohesive energy per atom of a perfect *hcp* lattice of Zr. Further, using climbing-NEB method^55^,^56^, we calculate the diffusion barrier of a vacancy and an interstitial toward the pristine and damaged GBs^40^. To calculate the diffusion barriers, we introduce a vacancy (an interstitial) at the desired lattice site as the initial state, then we select one of its nearest neighbors (nearest interstitial site) in the corresponding *hcp* unit cell as the final state, and construct the minimum energy path (MEP) for migration of the vacancy between two pre-defined states using multiple-replica algorithm^56^. Finally, via using the saddle point searching method^55^,^56^, we obtain the maximum value in each reaction coordinate. Of all investigated transitions, values that leads to the migration of the defects to the close vicinity of the pure GBs are selected^40^,^43^,^53^.

## Results and Discussion

### Primary radiation damage in *hcp* Zr with GBs

Using MD, we investigated the evolution of the collision cascades on ps timescale, and analyzed changes in the number of surviving defects, as well as the structure of residual defect clusters in the grain interiors, as a function of GB type and initial distance of the PKA from the GB plane. We began with the simulation of displacement cascades near symmetric tilt 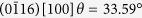, symmetric tilt 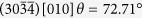 and twist 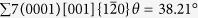, twist 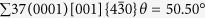 GBs in *hcp* Zr. The system was initially relaxed at 300 K, using the steepest descent method for 20 ps with a time step of 1 femtosecond (fs) to reach thermal equilibrium. For the rest of the simulation, microcanonical ensemble (NVE), with the system isolated without heat exchange was applied. Atoms on the lower grain, located at the center of the plane parallel to the GB, were selected as the PKAs^39^,^40^, and were given 3, 6 and 9 keV of kinetic energies with their velocity directed perpendicularly toward the GBs. The PKAs induced collision cascades and a smaller time step of 0.1 fs was used for an additional 3 ps to follow the melting phase of the cascades, after which the time step was increased back to 1 fs for about 60 ps to remove defects near the atomic GBs.

**Figure 2:**
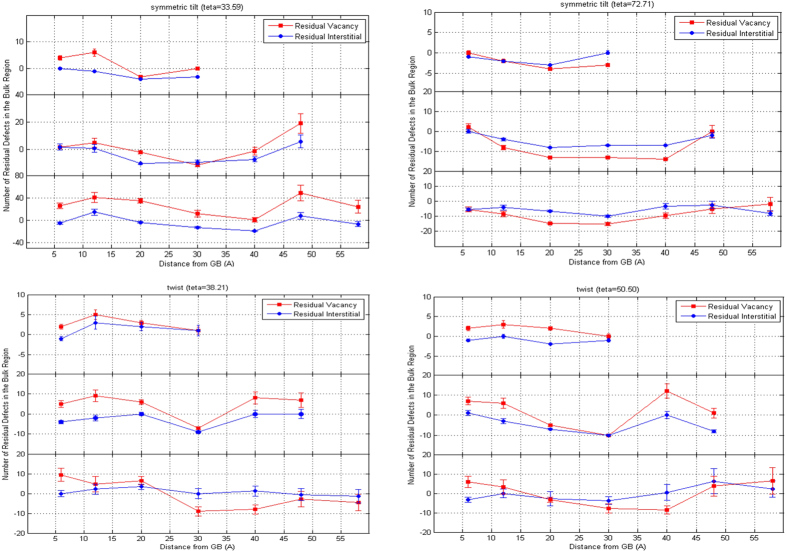
Number of interstitials and vacancies produced in the bulk region near each of the four GBs considered in this study, as a function of the initial distance of the PKA with 3, 6 and 9 keV of kinetic energies from the GB center. Values are relative to the number of defects produced in the equivalent cascades in a single crystal. The statistics are based on 8 simulations at each PKA distance and the error bars represent the standard error. In each figure, the upper, middle and lower curves illustrate the residual defect population for cascades initiated by 3, 6 and 9 keV PKAs, respectively.

The number of defects relative to the number of Frenkel pairs produced in a single crystal from cascades with the same PKA orientation and energy is presented in Fig. 2. Considering the fact that the number of radiation-produced defects (vacancies and interstitials) are essentially equal in single crystals^57^,^58^,^59^, it is obvious that the twist GBs, with qualitatively similar behaviors, result in aggregation of slightly more or equal number of vacancies compared with interstitials in the bulk region for all PKA energies, particularly when the cascades are initiated closer to or farther from the GB plane than at intermediate distances (approximately between 30 to 48 Å in our present study). The radiation-induced cascades slightly overlap with the boundaries, and most of the defects are produced in the bulk region, suggesting that in the edge-overlap, twist GBs are biased sinks for SIAs. Moreover, Fig. 2 exhibits an extended “interstitial-depleted” region near the boundaries for the cascades produced by 9 keV PKAs at 30 Å to 40 Å away from the GB plane, in which the cascades maximally overlap with the boundaries, and most of the defects are directly produced in the GB region, implying a biased absorption of vacancies over interstitials for high-energy PKAs at intermediate distances. Surprisingly, these results indicate that the sink efficiency of the twist structures strongly depends on the energy and distance of the PKAs from the GB plane, in addition to the GBs crystallographic orientation previously denoted by *Woo* in^47^.

Further, Fig. 2 illustrates that the number of residual vacancies is always equal or larger than the number of trapped interstitials in symmetric tilt 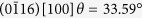, and suggests that the low-angle tilt GB is a strong sink for SIAs. Moreover, in this specific GB structure, the number of trapped vacancies in the bulk region increases with the increasing PKA energy. As previously reported for *fcc* Ni in^40^, an excess vacancy concentration during the collision cascade, only occurs because the GB absorbs interstitials before they can recombine with vacancies. On the contrary, the high-angle symmetric tilt 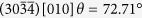 absorbs more vacancies and results in an interstitial-loaded bulk region. Further, the relative number of defects in vicinity of this GB structure is zero or negative for all PKA energies and distances, suggesting that the majority of defects in the grain interiors are annihilated through the in-cascade recombination of vacancies and interstitial atoms, and hence, the high-angle symmetric tilt GB acts as a weak sink for the irradiation-produced vacancies. Moreover, the trapped vacancies in the GB region are immobile on ps timescale. Overall, the results (Fig. 2) indicate that as the tilt angle of the GB plane increases, shifting from “semi-parallel” to “semi-perpendicular” relative to the basal planes, the symmetric tilt GBs show different behavior in terms of acting as sinks for the irradiation-produced defects, implying a strong dependency on the crystallographic orientation of the grains in a bi-crystal tilt structure. In either case, if we limit our investigation to low temperatures (300 K in our present study) where the thermal migration of defects can be neglected^40^, the defect content within the material during irradiation will be the aggregation of surviving vacancies or interstitial atoms in the bulk region, and accumulation of defects of opposite nature at the GB plane. It is worth noting that to have a better insight of the role of different atomic GBs on the defect production and evolution, the dependency of the primary damage to direction of the collision cascades should also be investigated. Overall, the results suggest that in a nano-crystalline Zr with a high density of mixed symmetric tilt GBs (low-angle tilt GB as a strong sink for SIAs, and the high-angle tilt GB as a biased absorber of vacancies), the number of radiation-produced defects in the bulk region will be maximally suppressed, which might lead to the enhanced healing of the nano-crystalline structure at longer timescales.

**Figure 3:**
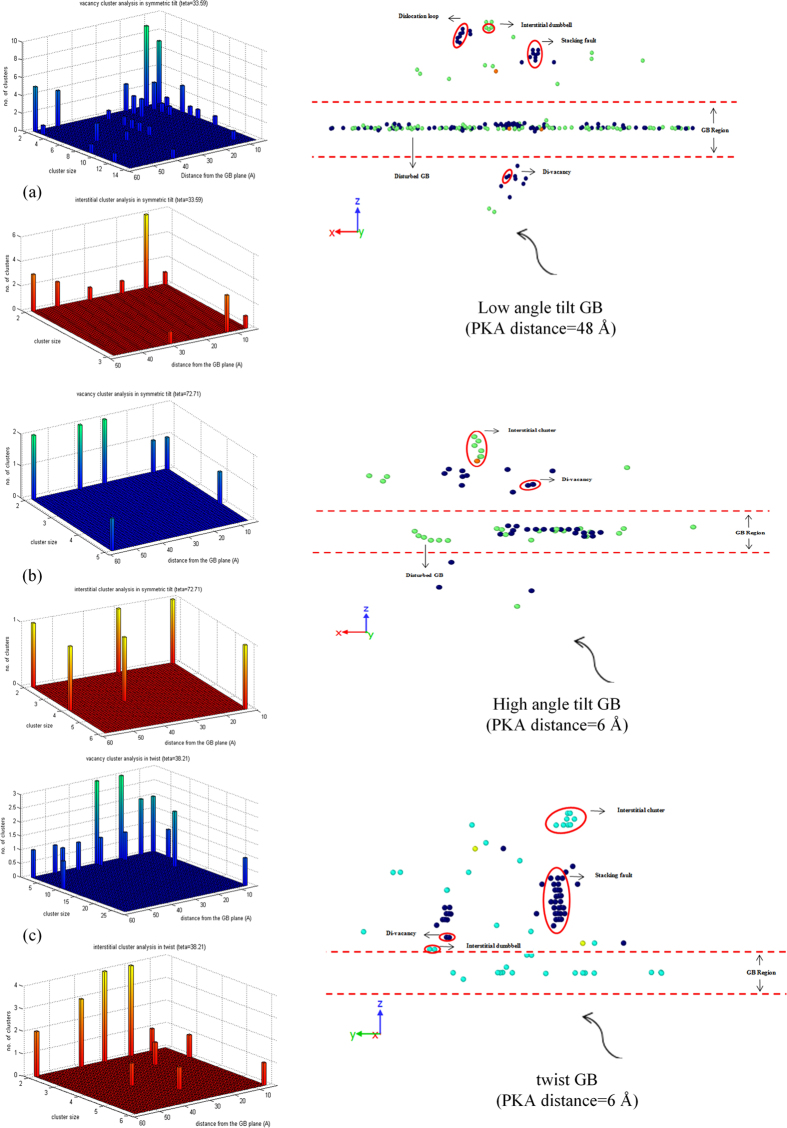
Analysis of the produced vacancy/interstitial clusters in the bulk region of the *hcp* Zr with atomic GBs; (**a**) symmetric tilt 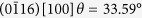; (**b**) symmetric tilt 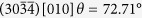; (**c**) twist 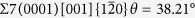. The analysis is conducted for the case with the most vacancy/interstitial-saturated bulk region, observed between 8 cascades simulation at each PKA distance. The inset of each curve is an example of the defect content in the grain interiors. In the inset figures, the axes of the reduced coordinates are: 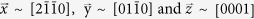.

To further investigate the residual defect content in the grain interiors, the produced vacancy/interstitial clusters, both in terms of population and size are analyzed and illustrated in Fig. 3. The analysis is conducted for the case with the most vacancy/interstitial-saturated bulk region, observed between 8 cascades simulation at each PKA distance with 9 keV of kinetic energy. The Fig. 3 is just an illustrative example to provide a better view over the deformed bulk structure in the presence of different atomic GBs. For the low-angle tilt structure, vacancy clusters such as stacking faults and vacancy-type dislocation loops, di-vacancies and interstitial dumbbells are formed in the bulk region, especially when the cascades are initiated closer to or farther from the GB plane (Fig. 3(a)). Further, the residual vacancies are immobile in the bulk region on ps timescale. As previously mentioned, the low-angle tilt structure is a sink for SIAs and thus, the GB region in the tilt structure is strongly disturbed due to the absorption of radiation-produced interstitials (inset of Fig. 3(a)). In contrast, the high-angle symmetric tilt GB facilitates the formation of interstitial dumbbells and clusters due to its bias over vacancies, although the residual defects in its grain interiors are in lower concentration compared with the other tilt structure (Fig. 3(b)). The GB region of the high-angle tilt structure is also locally disturbed due to the absorption of excess vacancies (inset of Fig. 3(b)), and production of interstitial atoms at the GB center. In case of the twist boundaries, the size and population of the vacancy/interstitial clusters depend on the distance of PKA from the GB plane, as shown in Fig. 3(c). Particularly, we observe the formation of vacancy clusters and di-vacancies in the cascades-GB slight overlaps (inset of Fig. 3(c)), as well as the formation of interstitial dumbbells and clusters as the cascade center maximally interacts with the boundaries. Interestingly, in all the simulations performed, GB regions of the twist structures only disturb due to the absorption of excess vacancies, and the trapped interstitials do not deform the GB structures, as shown in inset of Fig. 3(c). Overall, careful comparison of the defect clustering in bi-crystal *hcp* Zr has revealed that this phenomenon strongly depends on the microstructure of the atomic GBs, and how the presence of these interfaces modifies the spatial distribution of defects within the grain interiors.

**Figure 4:**
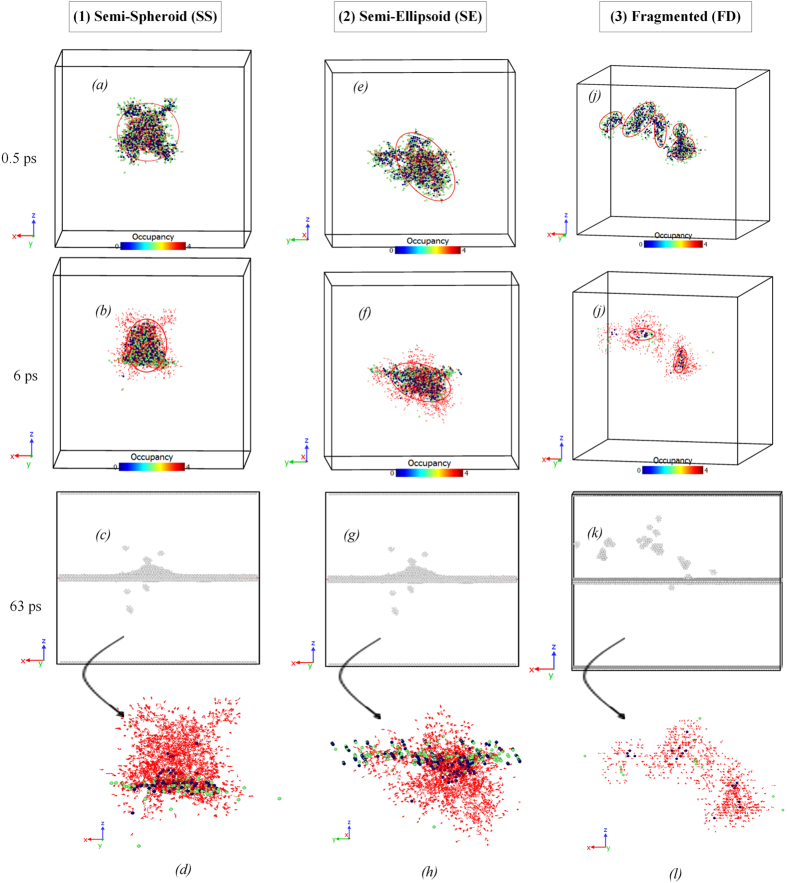
Geometric analysis of the primary cascades observed in MD simulations; (**a–d**) Semi-spheroid geometry; (**e–h**) Semi-ellipsoid geometry; (**i–l**) Fragmented distribution; (**a**,**e**,**i**) Thermal spike peak (t = 0.5 ps); (**b**,**f**,**j**) Middle of the cooling phase (t = 6 ps); (**c**,**g**,**k**) The damaged *hcp* crystal structure at the end of the cascade cooling phase (t = 63 ps), obtained using the adaptive common neighbor analysis (CNA) method^60^,^61^; (**d**,**h**,**l**) Snapshots of the relaxed cascade with the trajectories (red lines) of the interstitial atoms (t = 63 ps). In each figure, the axes of the reduced coordinates are: 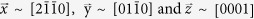.

To further elucidate defect-boundary interaction on the microscopic scale and provide a better understanding of the atomistic mechanisms that lead to negative or positive biases, we have analyzed the spatial distribution of defects within the primary cascades in *hcp* Zr with GBs. We have identified three distinct cascade geometries based on MD simulation results denoted as Semi-Spheroid (SS), Semi-Ellipsoid (SE) and Fragmented (FD) cascades, as shown in Fig. 4. In “Semi-Spheroid” geometry, the primary cascade extends along both {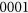} (basal) and {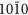} (prismatic) planes, which results in quasi-symmetric defect growth in the bulk region, as shown in Fig. 4(a). The “Semi-Ellipsoid” geometry, on the other hand, only extends along {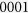} and/or {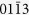} (compression) planes, leading to stretched exponential growth of the primary cascade (Fig. 4(e)). The defect content within SS/SE type’s cascades consists of the aggregated vacancies in the cascade core and dispersed interstitials in its outer layers, which shrinks in size as the in-cascade recombination of vacancies and interstitial atoms occur (Fig. 4(b,f)). The cascades initiated by 3 keV and 6 keV PKAs are predominantly SS/SE type (298 out of 320 simulations performed), which due to the low concentration of defects lead to the formation of residual Frenkel pairs, di-vacancies and interstitial dumbbells in the bulk region, depending on PKA distance and the microstructure of atomic GBs.

The 9 keV-induced cascades, however, rarely show symmetric/stretched defect growth in the MD simulations performed (59 out of 224 simulations at 9 keV PKA energy). But interestingly, the results have revealed that if the radical/semi-minor axis of SS/SE type’s cascades initiated by 9 keV PKAs, slightly overlaps with the boundaries, the residual defects (both interstitials and vacancies) will form a “finite cone” with its base on the GB plane (Fig. 4(c,d) and Supplementary Movie S1), while in maximal overlaps, the defect content will consist of dispersed interstitial dumbbells and aggregated di-vacancies, which eventually change into stacking faults in the close vicinity of the atomic GBs, as shown in Fig. 4(g,h) and Supplementary Movie S2. Further, due to the high concentration of defects and the resulting low-barrier “very short atomic displacements”^41^ within SS/SE types’ cascades, we observe a rather long thermal spike duration for these specific defect structures (~8 ps for 9 keV PKAs).

The more complex “Fragmented” distribution that frequently occurs in 9 keV-initiated cascades consists of two or more decentralized SS/SE type’s sub-cascades, each with a lower concentration of defects compared with the other two previously identified distributions, as illustrated in Fig. 4(i). Depending on PKA distance and the cascade growth direction, SS/SE sub-cascades separately extend both along {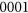} and either {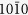} or {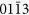} planes in the upper and/or lower grains and eventually, shrink in size due to the in-cascade recombination of vacancies and interstitial atoms with a rather short thermal spike duration (~4 ps for 9 keV PKAs) (Fig. 4(j)). The residual defect content of the fragmented distribution depends on the number and effective size of SS/SE sub-cascades and most often consists of locally dispersed di-vacancies and interstitial dumbbells within the grain interiors, as shown in Fig. 4(k,l) and Supplementary Movie S3.

Further investigation also reveals that the displacement cascade in symmetric tilt structures initiated by 9 keV PKA, tends to grow in semi-spheroid (semi-ellipsoid) form in slight overlaps, and shows a fragmented distribution as the cascade center gradually migrates toward the GB plane. In vicinity of the twist structures, on the other hand, the defects within the primary cascades are distributed in the fragmented form in the cascade-GB slight overlaps, and exhibit symmetric/stretched defect growth, when the cascade center maximally interacts with the boundary. Moreover, we find that the defect growth direction is independent of the degree of overlap between the boundary and the cascade damage region, and in layers close to the GBs, the trapped interstitials are thermally activated and can migrate between the upper and lower grains at 300 K. This indicates that the diffusion barrier of SIAs in the neighboring region of the atomic GBs is substantially reduced, which will be investigated in thermodynamics of defects. Overall, the results illustrate that the residual defect content and the clustering phenomenon in *hcp* Zr with GBs strongly depend on the spatial distribution of defects within the bulk region and how these atomic interfaces modify the energetic and kinetic behaviors of defects within the bi-crystal structures.

### Energetics and kinetics of vacancy and interstitial near pristine GBs

The MD simulations have clearly demonstrated that, depending on PKA distance and energy and microstructures of the atomic GBs, vacancies or interstitial atoms are absorbed at the boundaries, and defects of opposite nature are aggregated in the bulk regions. In order to show energetically and kinetically whether a vacancy or an interstitial has a tendency to migrate into the GB, we calculate the defect formation energies and diffusion barriers at various locations in the GB structures^40^. During the calculation of formation energies to study the energetics of defects in the presence of GBs, initially, we created one vacancy or an interstitial in the simulation box^39^,^40^,^43^. To introduce a vacancy, an atom at a particular site, within 15 Å of the boundary is removed and then the system is minimized using the steepest descent method. While for the introduction of an interstitial, we initially search for a perfect *hcp* unit cell for that particular site (if it is found), then in the “octahedral” site, an additional atom is inserted. If no such perfect unit cell exists, e.g., in the nearest layer of the GB where the coordinate number is lower compared with that in the bulk^40^, an atom is created at the center of two nearest neighbors. Finally, the simulation cell is relaxed, using the same method as in the case of a vacancy^40^.

**Figure 5:**
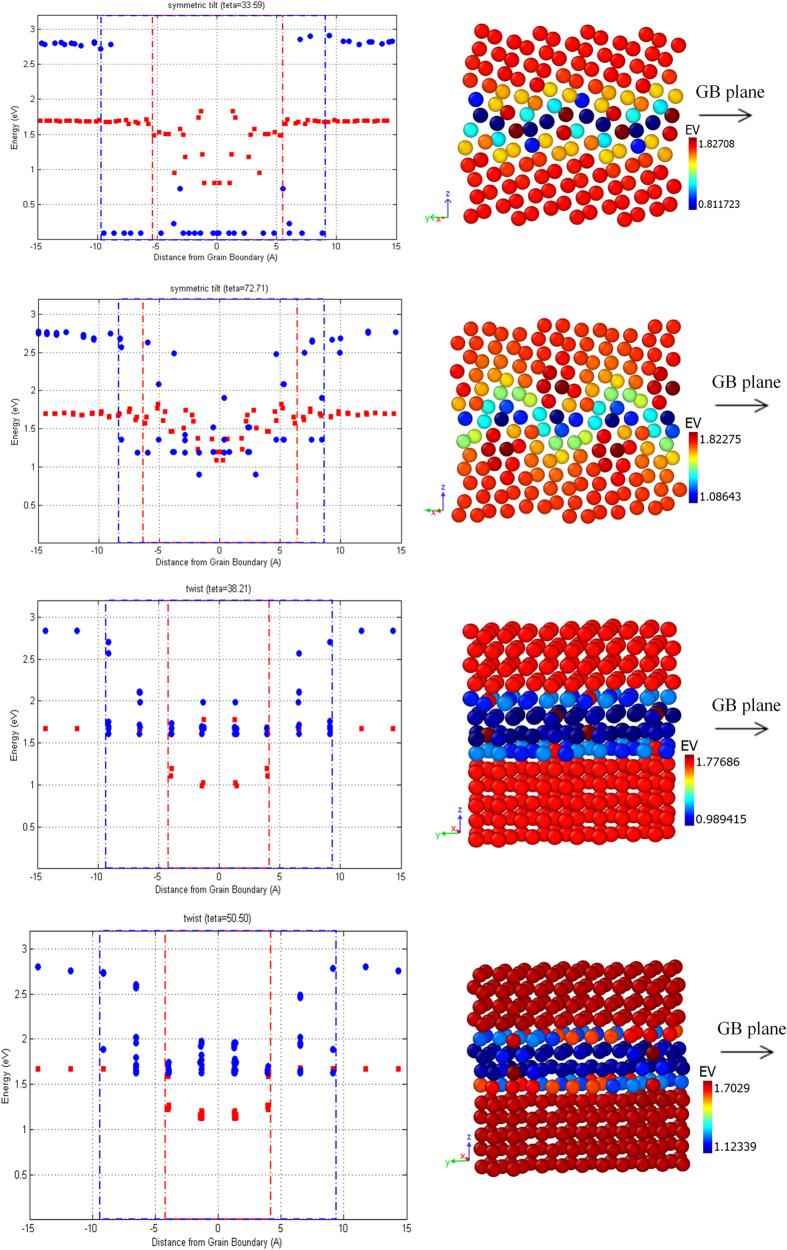
(**a**) The vacancy/interstitial formation energy profiles of four GB structures considered in the present study. In each figure, the blue circles and red squares exhibit the interstitial and vacancy formation energies, correspondingly. The blue and red dashed lines demonstrate the GBs energetic influence range, in which the defects formation energies decrease to less than 80% of s bulk values; (**b**) Two-dimensional (2D) vacancy formation energy profile (E_V_) of the *hcp* Zr with four different atomic GBs. In each figure, the axes of the reduced coordinates are: 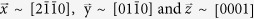.

The formation energy profile of vacancies (E_v_) and interstitials (E_I_) near the atomic GBs in *hcp* Zr is shown in Fig. 5(a). In each curve, the red and blue dashed lines illustrate the energetic influence range of the atomic GBs for vacancies and interstitials, respectively. Further, the two dimensional (2D) mapping of vacancy formation energy in the GB structures is also demonstrated in Fig. 5(b). It is obvious that the defect formation process is always endothermic. The formation energy generally decreases as the defects migrate from the bulk to the GB plane, which indicates an attractive interaction between defects and the pristine boundaries. This means that, it is energetically favorable for a vacancy or an interstitial atom to form and reside at the GB, consistent with previous reports for cubic metals in^39^,^40^,^43^,^53^. Considering the influence range and the sink strength of the atomic GBs (decrease in E_v_ and E_I_ relative to the bulk values), we find that the low-angle symmetric tilt structure, with an energetic interaction width of 18.8 Å for interstitials and a rather large reduction of E_I_ (~98%) in vicinity of the boundary, shows a very strong attractive interaction that might be the reason to observe biased absorption of interstitials over vacancies in this specific GB structure. The relatively large reduction in E_I_ implies that the system energy is decreased more through interstitials occupying GB sites, rather than vacancies. Further, Fig. 5(b) demonstrates that at the GB center, atomic sites appear symmetrically that have E_V_ close to or even higher than the bulk values, implying that the low-angle symmetric tilt boundary may not provide pathways for vacancy diffusion, as previously reported in^46^. On the other hand, the high-angle symmetric tilt GB exhibits a larger energetic influence range for vacancies (~12.5 Å) with symmetrically extended sites with low values of E_V_ in the bulk (Fig. 5(b)), and a rather smaller reduction of E_I_ (~61%) and E_V_ (~36%) in close vicinity of the boundary in comparison with the other tilt structure, which can explain the preferential absorption of vacancies over interstitials observed in MD simulations, and relatively the weaker sink strength of this atomic GB.

Moreover, Fig. 5(a) illustrates a qualitatively identical behavior of defect energetics in close vicinity of the twist boundaries, with an attractive interaction width of 8.4 Å (~18.9 Å) for vacancies (interstitials), and rather small reduction of E_I_ (~34%) and E_V_ (~41%) at the GB center, indicating that energetically, the twist structures can act as defect sinks for interstitial atoms with relatively lower sink strength. But, the MD results illustrated that the cascades initiated by 9 keV PKAs at intermediate distances would result in an interstitial-depleted GB regions, which is in contrast with the energetic properties of defects. Further investigation of the 2D mapping (Fig. 5(b)) demonstrates that the E_V_ decreases to its minimum value when a vacancy is formed in the layers very close to the boundary, implying that if the cascade center maximally overlaps with the GB plane and a large fraction of the PKA energy is dissipated in this region, a high concentration of vacancies will form, and during the cooling phase, due to the in-cascade recombination of defects will result in an interstitial-depleted region near the GB center with the excess vacancies trapped at the boundaries, which explains the observed contradiction. Eventually, the results demonstrate that in terms of energetic behavior, the atomic GBs indeed serve as sinks for the radiation-produced defects with a preference for either vacancies or interstitial atoms, depending on the GB type, the crystallographic orientation and PKA energy and distance, which in turn, determines the distribution of defects within the cascade damage region.

**Figure 6:**
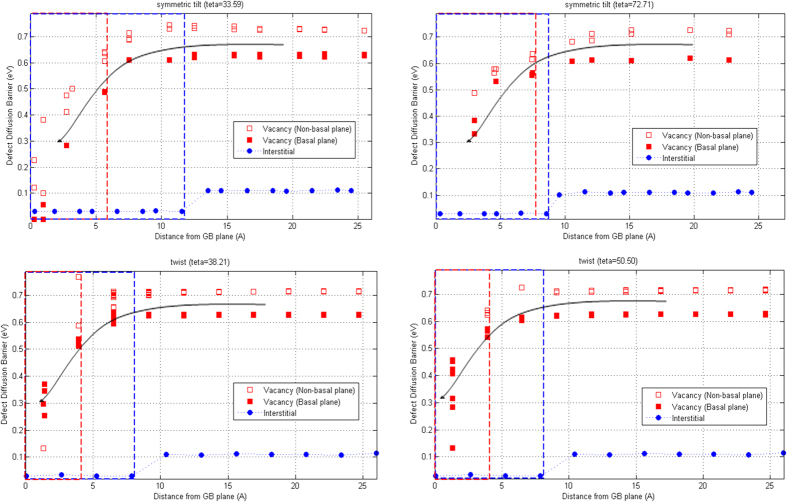
Diffusion barriers of a vacancy and an interstitial atom near the pristine GBs considered in the present study. Defect diffusion barriers are plotted as a function of distance from a pristine GB. The arrows are drawn only as a guide to the eye. In each curve, the red and blue dashed lines illustrate vacancy and interstitial kinetic influence ranges, respectively.

Next, we study the kinetic behavior of defects migrating from the bulk to close vicinity of the atomic GBs. Using the climbing-NEB method, the diffusion barrier of a vacancy and an interstitial atom is calculated and shown in Fig. 6. Two nearest neighbors, within all sampled defect configurations completely relaxed for calculating defect formation energies above, were used as the initial and final states^40^. Between the two states 24 replicas are inserted. The red and blue dashed lines in Fig. 6 demonstrate the kinetic influence range of the GB structures for vacancies and interstitials, respectively, where the calculated barriers decrease to less than 80% of the bulk values. Fig. 6 illustrates that the diffusion barriers are significantly reduced when defects migrate to the close vicinity of the atomic GBs. The vacancy barrier in the bulk grain is approximately 0.62 eV (0.71 eV) for migration in non-basal (basal) plane, and reduces to ~0 eV, ~0.33 eV and ~0.16 eV in layers close to the boundary in symmetric tilt 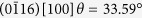, symmetric tilt 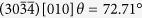 and twist GBs, respectively. The interstitial diffusion barrier, on the other hand, with 0.13 eV in the bulk decreases to less than 0.01 eV, when an interstitial diffuses to the vicinity of the symmetric tilt and twist structures, and within a certain range of the GBs (blue dashed lines in Fig. 6) interstitials are trapped at the boundaries via low-barrier processes.

Considering the kinetic influence range and the relative decrease in diffusion barriers at the GB plane, we find that the low-angle symmetric tilt structure illustrates a wider kinetic interaction width (~12.1 Å) for the interstitials, indicating that the SIAs produced at farther distances from the GB can move toward the boundary via low-barrier processes. This can be another explanation to observe the biased absorption of interstitials over vacancies in this specific GB structure. Therefore, energetically and kinetically symmetric tilt 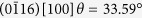 GB can act as a defect sink with a preference for SIAs, due to the remarkably large reduction of E_I_, an extremely low defect diffusion barrier near the boundaries and a much wider energetic and kinetic influence ranges for the interstitial atoms. Further, the vacancy migration barrier in the low-angle tilt structure decreases to 0 eV at the GB center, implying that the vacancies (if formed due to the absorption of excess interstitials) can move at the GB plane via barrier-free processes. Thus, the preferential absorption of interstitials at the GB and the barrier-free migration of vacancies in the boundary region can explain the disturbance of the low-angle tilt GB observed in MD simulations. On the other hand, the high-angle symmetric tilt structure with a larger interaction width (~7.6 Å) for vacancies and a rather smaller kinetic influence range (~8.3 Å) for interstitial atoms facilitates the formation of extended range of PKA distances over which the GB results in significantly reduced residual vacancy numbers in the bulk region (Fig. 2). Moreover, the vacancy diffusion barrier in the symmetric tilt 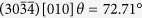 structure decreases to 0.33 eV in close vicinity of the GB plane, with an activation temperature of (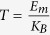, where 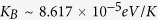 is Boltzmann constant and 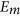 is the defect migration barrier^53^) ~3829.5 K, indicating that in the low temperatures (300 K in the present study), the trapped vacancies will be immobile in the GB region, consistent with the MD results.

Further, we find that the twist structures exhibit qualitatively similar kinetic behaviors with an interaction width of 4.1 Å (~7.2 Å) for vacancies (interstitials), indicating that kinetically the twist GBs can act as defect sinks for the interstitial atoms. In the previous section, we provided an explanation to observe the extended interstitial-depleted boundary region in the cascade-GB maximal overlaps near the twist boundaries in terms of the defect energetics (Fig. 5). Next, we discuss the possible reasons for the twist structure to act as a sink with a preference for interstitial atoms, when the cascade center slightly interacts with the GB region at high PKA energies. The MD simulations illustrate that in the cascade-GB slight overlaps near the twist boundaries, the defects are distributed in the fragmented form. Most of the defects are produced in the bulk region in the form of SS/SE sub-cascades and annihilated through the subsequent in-cascade recombination of point defects. So, the residual defect content for part of the cascade that does not interact with the boundaries will be an equal number of vacancies and interstitials in the bulk grain. Further, only a small fraction of the radiation-produced defects form in the region close to the GB center. Considering the kinetic interaction width and the decrease in the defect migration barriers, we find that the twist structures facilitate the diffusion of SIAs toward the GB plane with a barrier of 0.007 eV (activation temperature ~81.3 K), while the vacancies will be immobile in the region close to the boundary due to their higher migration barriers (~0.16 eV) and activation temperatures (~1856.7 K), leading to an interstitial-saturated GB surrounded by excess vacancies in the bulk region, consistent with MD simulations.

### Energetics and kinetics of vacancies near the damaged GBs

The energetic and kinetic behaviors of a vacancy and an interstitial exhibited that the atomic GBs in *hcp* Zr act as sinks for the irradiation-produced defects, consistent with MD simulations. Moreover, independent of the sink efficiency and strength of the GBs, the dynamic results clearly demonstrated that the boundaries would start to deform, either due to the absorption of SIAs or vacancies and the subsequent production of the interstitial atoms at the GB center, indicating that at the end of the cooling phase, the primary cascades would result in an interstitial-loaded (damaged) GB on ps timescale. In order to specifically study the influence of the damaged GBs on the formation and evolution of the excess vacancies in the bulk region, we intentionally loaded the GBs with 5 and 10 interstitials at 0 K. To create damaged GBs, we used the same method described in^40^, and further investigated the energetics and kinetics of the vacancies near the interstitial-loaded GBs.

**Figure 7:**
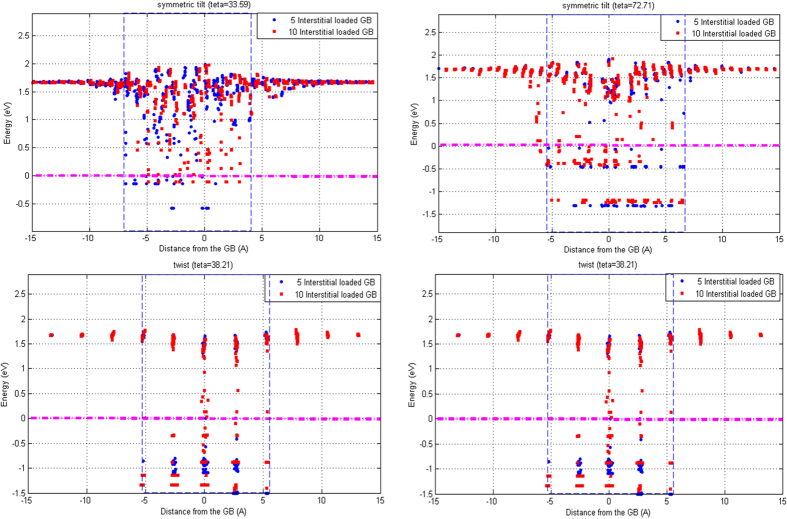
Vacancy formation energy profile of the GBs loaded with 5 (blue colored circles) and 10 (red colored squares) interstitials. In each figure, the pink dashed line separates the sites with positive and negative formation energies, and the blue dashed line demonstrates the energetic influence range of the damaged GBs.

Figure 7 illustrates the formation energy profile of a vacancy near the interstitial-loaded GBs. It is obvious that in comparison with the pristine boundaries, the E_V_ is significantly reduced, e.g., at the GB center, we observe a rather large reduction of 0.7 eV, 1.1 eV and 0.95 eV for the low-angle tilt, high-angle tilt and twist structures, respectively. This implies that the presence of excess interstitials at the boundaries will facilitate the defect formation process in the close vicinity of the damaged GBs, consistent with previous reports for cubic metals in^40^,^43^,^53^. Moreover, Fig. 7 demonstrates that in the nearest neighbors of the trapped interstitials, sites with negative vacancy formation energies appear, indicating an exothermic process to create a vacancy at one of these sites and the resulting reduction in the system energy. Further investigation also reveals that the vacancies at these sites are unstable and tend to spontaneously recombine with the trapped interstitials at the GBs, in agreement with previous reports in^40^,^53^. Also, we find that the energetic interaction width of the defect-loaded structures (the blue dashed lines in Fig. 7) significantly changes, and the appearance of the sites with near-zero or negative E_V_ depends on how the excess interstitial atoms accommodate at the GB plane. For example, presence of the trapped interstitials results in an extended interaction width in the lower grain for symmetric tilt 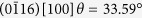 GB, while the defect-loaded symmetric tilt 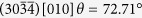 structure exhibits a wider energetic influence range in the upper grain, as shown in Fig. 7.

**Figure 8:**
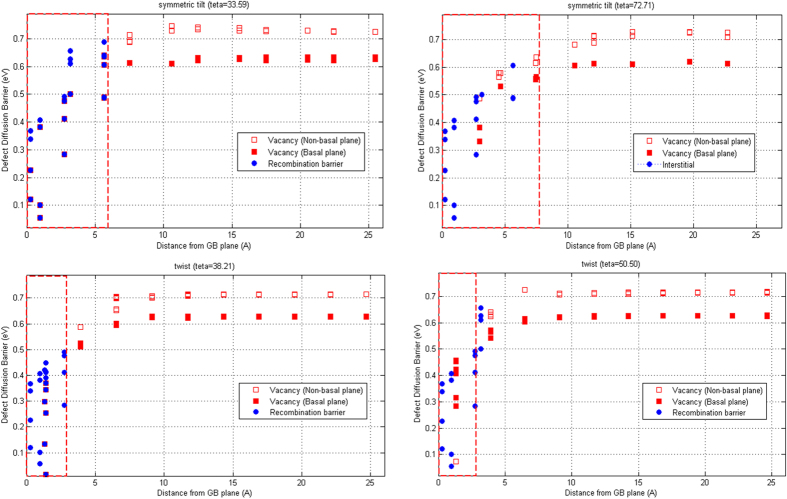
Vacancy diffusion and annihilation barriers as a function of distance from the GBs loaded with five interstitials (the damaged GB structures). In each curve, the red dashed line represents the kinetic interaction width of the damaged GB.

Finally, in order to investigate the kinetic behavior of the vacancies in the neighboring region of the interstitial-loaded GBs, we calculate the migration barrier of a vacancy diffusing from the bulk to the GB using the climbing-NEB method, as shown in Fig. 8. We define the recombination barrier as the one that a vacancy, accommodated at the nearest neighbors of the trapped interstitials, overcomes before diffusing and thus, annihilating the interstitials^40^. The comparison of Figs 8 and 6 reveals that the migration barriers are also significantly reduced in the close vicinity of the defect-loaded GBs, indicating that in the presence of thermally assisted events, the damaged GBs will facilitate the migration of excess vacancies in the bulk grain toward the GB plane, in agreement with^40^,^43^,^53^. Further investigation also demonstrates that the annihilation barriers (the blue colored circles in Fig. 8) strongly depend on the distance and orientation of a vacancy and the trapped interstitials and eventually, lead to rather large recombination barriers in certain orientations and distances. Moreover, the kinetic interaction width of the defect-loaded structures do not significantly change in comparison with the pristine GBs, which has a simple physical explanation as discussed in our previous report on *fcc* Ni^40^. *Shao et al.*^35^ reported that the loaded interstitials will migrate toward the GBs, and reside in its close vicinity at sites with the lowest interstitial formation energy. Thus, the presence of defects at the GB plane only influences the migration of excess vacancies, as they diffuse and accommodate at the nearest neighbors of the trapped interstitials and may not change the kinetics of vacancies at farther distances from the GB center^40^.

## Conclusions

A combination of MD, MS and climbing-NEB method was used to investigate the influence of different atomic GBs on the production and the subsequent evolution of the irradiation-induced point defects in *hcp* Zr on ps timescale, and the following conclusions were drawn:

1. The twist GBs exhibit similar behavior in terms of acting as sinks for the irradiation-produced defects, with qualitatively identical energetic and kinetic properties. The sink efficiency and the sink strength of these structures depend on the PKA energy and its distance from the GB plane, in addition to the crystallographic orientation of the grains within the bi-crystals, previously denoted in theory of irradiation in non-cubic metals.
2. The low-angle symmetric tilt structure acts as a strong sink for SIAs, while the high-angle symmetric tilt GB absorbs vacancies with rather smaller sink strength. In either case, the defect content within the material during irradiation will be the aggregation of surviving vacancies or interstitial atoms in the bulk region, and accumulation of defects of opposite nature at the GB plane. The MD simulations also exhibit that the GBs are disturbed either due to the absorption of SIAs or vacancies and the subsequent production of interstitials at the GB center.
3. The results suggest that in a nano-crystalline Zr with a high density of mixed symmetric tilt GBs (low-angle tilt GB as a strong sink for SIAs, and the high-angle tilt GB as a biased absorber of vacancies), the number of radiation-produced defects in the bulk region will be maximally suppressed, which might lead to the enhanced healing of the nano-crystalline structure at longer timescales.
4. We identify three distinct cascade geometries based on MD simulations, denoted as semi-spheroid (SS), semi-ellipsoid (SE) and fragmented (FD) distributions. The cascades initiated by low-energy PKAs are predominantly in SS/SE form, and depending on the PKA distance and the GB type, we observe either SS/SE or FD distributions for 9 keV PKA cascades. Further, the clustering of the residual defects near different GBs depends on how these atomic interfaces modify the spatial distribution of defects within the cascade damage region.
5. To further elucidate the role of atomic GBs in modifying defect evolution in hcp Zr, the dependency of the primary damage to direction of the collision cascades should also be investigated.
6. The energetic and kinetic results demonstrate that the pristine GBs serve as sinks for the radiation-produced defects with a preference for either vacancies or interstitial atoms, depending on the GB type, the crystallographic orientation and PKA energy and distance, which in turn, determines the distribution of defects within the cascade damage region.
7. The damaged GBs, on the other hand, facilitate the formation and migration of excess vacancies in the bulk region toward the boundaries. We observe the formation of sites with negative E_V_ near the interstitial-loaded GBs, and that the vacancies in these sites are unstable and tend to spontaneously recombine with the trapped interstitials at the GB center. Moreover, we find that the appearance of sites with negative E_V_ depends on how the excess interstitials accommodate at the GB center.

## Additional Information

**How to cite this article**: Arjhangmehr, A. and Feghhi, S. A. H. Irradiation deformation near different atomic grain boundaries in α-Zr: An investigation of thermodynamics and kinetics of point defects. *Sci. Rep.* **6**, 23333; doi: 10.1038/srep23333 (2016).

## Supplementary Material

Supplementary Information

Supplementary Movie S1

Supplementary Movie S2

Supplementary Movie S3
