# Supplementary material for: Irradiation deformation near different atomic grain boundaries in α-Zr: An investigation of thermodynamics and kinetics of point defects
Source: Sci Rep. 2016 Mar 23;6:23333. doi: 10.1038/srep23333 (PMC4804209; doi:10.1038/srep23333)
Supplement: Supplementary Information [file srep23333-s1.pdf]

Irradiation deformation near different atomic grain boundaries in α-Zr: An investigation of thermodynamics and kinetics of point defects

A. Arjhangmehr[1] & S. A. H. Feghhi[*1]

[1] Department of Radiation Application, Shahid Beheshti University G.C, Tehran, IRAN
ms.arjangmehr@gmail.com; a.feghhi@gmail.com

Supplementary Movie S1. The cooling phase of the semi-spheroid cascade geometry identified during the MD simulations. It is obvious that if the radical/semi-minor axis of SS/SE type's cascades initiated by 9 keV PKAs, slightly overlaps with the boundaries, the residual defects (both interstitials and vacancies) will form a "finite cone" with its base on the GB plane.

Supplementary Movie S2. The cooling phase of the semi-ellipsoid cascade geometry identified during the MD simulations. In maximal overlaps between the radical/semi-minor axis of SS/SE type's cascades initiated by 9 keV PKAs with the boundaries, the defect content will consist of dispersed interstitial dumbbells and aggregated di-vacancies, which eventually change into stacking faults in the close vicinity of the atomic GBs.

Supplementary Movie S3. The cooling phase of the fragmented cascade geometry identified during the MD simulations. The residual defect content of the fragmented distribution depends on the number and effective size of SS/SE sub-cascades and most often consists of locally dispersed di-vacancies and interstitial dumbbells within the grain interiors.
